# Both Drosophila matrix metalloproteinases have released and membrane-tethered forms but have different substrates

**DOI:** 10.1038/srep44560

**Published:** 2017-03-16

**Authors:** Kimberly S. LaFever, Xiaoxi Wang, Patrick Page-McCaw, Gautam Bhave, Andrea Page-McCaw

**Affiliations:** 1 Dept. of Cell and Developmental Biology, Vanderbilt University School of Medicine, Nashville, TN, USA; 2 Center for Matrix Biology, Vanderbilt University Medical Center, Nashville, TN, USA; 3 Program in Developmental Biology, Vanderbilt University, Nashville, TN, USA; 4 Dept. of Molecular Physiology and Biophysics, Vanderbilt, University School of Medicine, Nashville, TN, USA; 5 Dept. of Medicine, Vanderbilt University Medical Center, Nashville, TN, USA; 6 Dept. of Cancer Biology, Vanderbilt University School of Medicine, Nashville, TN, USA.

## Abstract

Matrix metalloproteinases (MMPs) are extracellular proteases that can cleave extracellular matrix and alter signaling pathways. They have been implicated in many disease states, but it has been difficult to understand the contribution of individual MMPs, as there are over 20 MMPs in vertebrates. The vertebrate MMPs have overlapping substrates, they exhibit genetic redundancy and compensation, and pharmacological inhibitors are non-specific. In contrast, there are only two MMP genes in Drosophila, DmMmp1 and DmMmp2, which makes Drosophila an attractive system to analyze the basis of MMP specificity. Previously, Drosophila MMPs have been categorized by their pericellular localization, as Mmp1 appeared to be secreted and Mmp2 appeared to be membrane-anchored, suggesting that protein localization was the critical distinction in this small MMP family. We report here that products of both genes are found at the cell surface and released into media. Additionally, we show that products of both genes contain GPI-anchors, and unexpectedly, that GPI-anchored MMPs promote cell adhesion when they are rendered inactive. Finally, by using new reagents and assays, we show that the two MMPs cleave different substrates, suggesting that this is the important distinction within this smallest MMP family.

Matrix metalloproteinases are extracellular proteases that cleave a variety of substrates including extracellular matrix components and regulators of extracellular signaling^1^,^2^,^3^. The first member of this protease family was identified as a biochemical activity from the histolyzing tissues of tadpoles in 1962^4^, and the biochemistry of these enzymes has been intensively studied for over 50 years since then. The MMP domain structure is conserved across multicellular eukaryotes, including plants like Arabidopsis, and animals from Hydra to Drosophila to humans. Because they are proteases, most MMP functions are understood to reside in the catalytic domain, which contains an active-site zinc ion. All MMPs are synthesized in zymogen form, with an autoinhibitory pro-domain that renders the enzyme inactive until the pro-domain is cleaved or destabilized. In nearly all MMPs, the catalytic domain is connected by a flexible hinge to a four-bladed beta-propeller hemopexin domain, important for substrate recognition. Within the mammalian MMP family, 7 MMPs are insoluble, tethered to the extracellular face of the plasma membrane by a transmembrane domain or a GPI anchor, and the remaining 17 MMPs are soluble secreted proteins^1^,^5^,^6^. The association of MMPs with tumor progression and metastasis has driven enormous clinical interest in these proteases^7^.

With the possibility of developing inhibitor strategies for the clinic, it has been important to delineate the functions of individual MMPs, as well as classes of MMPs, with respect to health and disease. A few mammalian MMPs have been extensively investigated using biochemical approaches, with the goals of understanding mechanisms of enzyme activation, inhibition, and substrate specificity. Yet because of the large number of MMPs – 24 in humans – it has not been possible to analyze all family members in great detail. Genetic analysis of mutants has been more comprehensive, as most MMPs have been knocked out in mice^1^,^8^,^9^,^10^,^11^. However, there is clear evidence of recent gene duplications within the MMP family, and redundancy and compensation have been observed between MMP family members in knockout mice^12^,^13^,^14^,^15^. These complications make it difficult to interpret the mild phenotypes of some MMP mutants. How then do the MMPs differ? Why are there so many? These questions have bedeviled the field for decades.

The fruitfly *Drosophila melanogaste*r has just two MMP genes, *DmMmp1* and *DmMmp2*^16^,^17^,^18^. Drosophila is an attractive organism for genetic analysis of MMP function because of the small family size, the sophisticated genetic techniques available, and the surprising degree of biological conservation between flies and mammals. The expression patterns of the two fly MMPs are very different, and genetic analysis with over a dozen MMP alleles, including conditional temperature-sensitive alleles, has demonstrated some distinct and some shared functions for each MMP. For example, *Mmp1* is required for tube elongation and circadian rhythm^19^,^20^, *Mmp2* is required for Wnt signaling regulating stem cells and for ovulation^2^,^21^, each MMP is required for motorneuron axon outgrowth and epidermal wound healing^22^,^23^, and both MMPs act redundantly in blood clotting and degrading basement membrane at metamorphosis^23^,^24^. Thus, in this simplified system, it is clear that each MMP is required for some separate functions and they work together for others. But the question persists – how are these two MMPs different from each other and why are there two of them? It has previously been reported that Mmp1 is secreted and Mmp2 is membrane-tethered, suggesting that the chief difference between them is their distinct cellular localization^17^,^18^,^23^. However, recent genome annotation has identified an *Mmp1* cDNA that encodes a GPI-anchor domain^25^, casting doubt on cellular localization as an evolutionary rationale for multiple MMP genes.

Despite its advanced genetic techniques, Drosophila has not been a powerhouse for biochemical analysis because of the small size and cellular complexity of its tissues. Thus, the biochemical analysis of fly MMPs has lagged. In this report we begin to rectify the imbalance by comprehensively characterizing the biochemistry and cell biology of the products of the two fly MMP genes in an insect cell culture system. We find that the difference between the two fly genes is not an essential difference in their cellular localization, as both Mmp1 and Mmp2 can be membrane-tethered and secreted. Rather, we find that the essential difference between the two fly MMPs lies in their substrate-specificity.

## Results

### Alternative Splicing Generates Several MMP Isoforms

**Figure 1:**
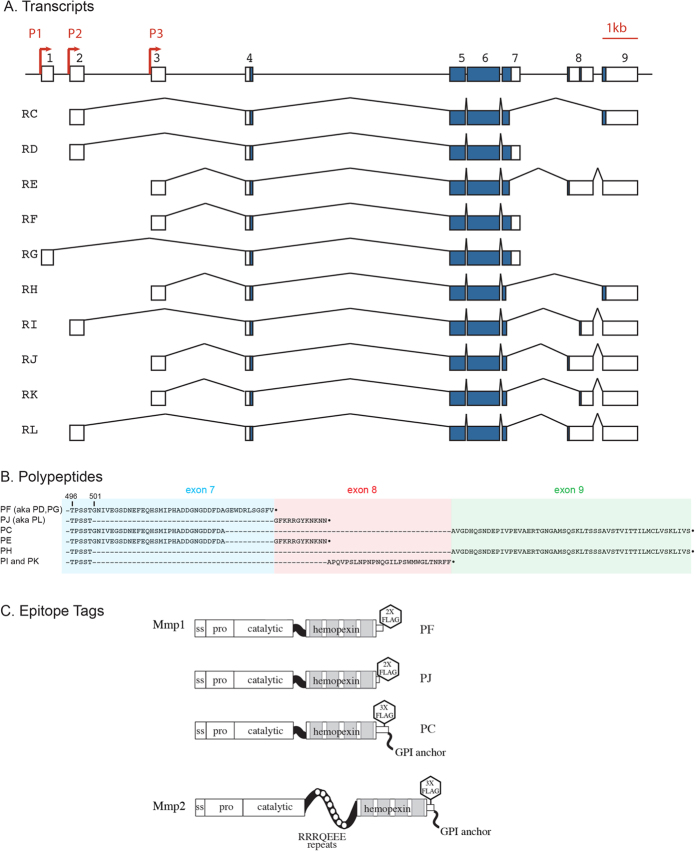
Many isoforms of DmMmp1 are predicted. (**A**) Genome organization of all 10 predicted splice forms of DmMmp1 identified by Flybase. Alternative splicing is confined to the ends: at the 5′ end, three alternative promoters determine the first non-coding exon, and at the 3′ end, alternative splicing affects the last three coding exons, 7, 8, and 9. Flybase nomenclature uses R to designate transcripts (RNA), e.g. Mmp1-RC. (**B**) 3′ alternative splicing generates six predicted polypeptides from the 10 predicted DmMmp1 transcripts. All six predicted polypeptides are identical for the first 500 amino acids and vary in the last 12–84 residues as shown. Flybase nomenclature uses P to designate protein products, e.g. Mmp1-PC is encoded by Mmp1-RC. (**C**) Domain structure and epitope placement for the MMPs used in this study.

The two Drosophila MMP genes reside in distinct regions of the genome at cytological bands 46A (*DmMmp2*) and 60D (*DmMmp1*), with more than half of chromosome arm 2R between them, and phylogenetic analysis demonstrates that they are not recent duplications^18^. Despite the small MMP gene number, 10 alternatively spliced Mmp1 transcripts have been annotated by Flybase and the Drosophila Genome Project, Mmp1-RC through Mmp1-RL, (“R” for “RNA”). These transcripts are initiated from three different promoter sites and are predicted to encode six distinct polypeptides. Some transcripts encode identical proteins (e.g., Mmp1-PD, -PF, and -PG encode the same protein, “P” for “Polypeptide”), but differ in the size and sequence of their UTRs (Fig. 1A,B). Most interestingly, the proteins Mmp1-PC and Mmp1-PH are predicted to encode GPI-anchored proteins. All six predicted Mmp1 proteins are identical for the first 500 amino acids including all of the catalytic and hemopexin domains. The only variable region is the C-terminal domain, which ranges from 12–84 residues in length. It is unclear how much the alternatively spliced isoforms of Mmp1 contribute to MMP function in Drosophila. Only three of the six predicted Mmp1 protein isoforms are represented by full-length cDNAs (Flybase): Mmp1-PF and Mmp1-PJ, which are predicted to be secreted, and Mmp1-PC, with a predicted GPI anchor. Thus we confined our analysis to these three isoforms of Mmp1.

For Mmp2, although three transcripts have been annotated in Flybase, cDNA evidence exists for only one of these transcripts, Mmp2-RB. The other two potential transcripts predict proteins with altered N-termini lacking pro-domains and predicted to localize inside the cell. These hypothetical transcripts will not be further considered in this paper. We confined our analysis to the Mmp2-RB/PB transcript and protein, which has all the hallmarks of a canonical MMP, including a signal sequence and pro-domain. This isoform has been previously described^17^,^18^.

To investigate the functional diversity within the uniquely small fly MMP family^26^, we expressed full-length cDNAs with FLAG-tags in insect S2 cells. A cDNA encoding Mmp1-PC was identified by Flybase, and we previously identified cDNAs encoding Mmp1-PF, Mmp1-PJ, and Mmp2 by library screening^18^. (Although we previously denoted Mmp1-PF and Mmp1-PJ as Mmp1.f1 and Mmp1.f2 respectively, we utilize Flybase nomenclature here for correspondence with the public databases.) We inserted FLAG epitopes at the C-termini of Mmp1-PF and Mmp1-PJ, as the N-terminus is expected to be cleaved during proteolytic activation. However, we were challenged to find a placement of the FLAG epitope for the predicted GPI-anchor containing proteins, Mmp1-PC and Mmp2, as the C-terminus would be cleaved if these proteins were glypiated. Inserting FLAG after the N-terminal furin recognition sequence caused both enzymes to be inactive (data not shown). We generated tagged Mmp2 constructs with five different insertion sites and assessed them for stability and localization in S2 cells (see Methods for tag locations). In intact flies, an Mmp2 fusion protein with GFP inserted after S710 rescued an *Mmp2* mutant, assuring that the tag does not interfere with function^2^. Moreover, insertion of a FLAG epitope after S710, C-terminal to the hemopexin domain and but proximal to the GPI-anchor signal sequence, generated a stable and catalytically active protein (see below). We copied that FLAG placement into Mmp1-PC, inserting FLAG after the hemopexin domain after amino acid E548, where it does not disrupt any known functional domains or modifications. Figure 1C shows schematics of the four proteins we expressed.

### A monoclonal antibody can distinguish between splice forms of Mmp1

**Figure 2:**
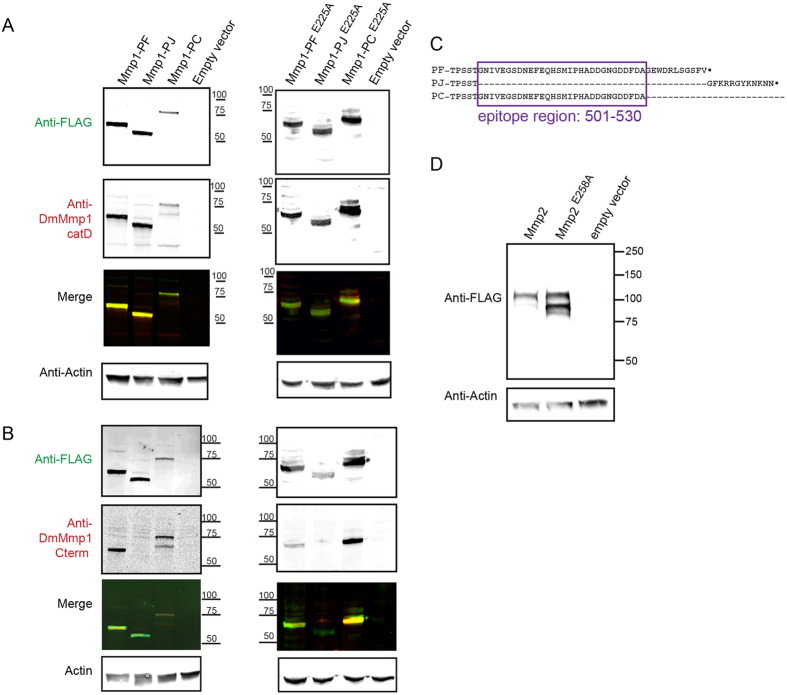
Full-length Drosophila MMPs and their inactive mutants are expressed stably in insect cells. (**A,B**) Western blots of three Mmp1 splice forms from Fig. 1C and corresponding catalytically inactive mutants (E225A) expressed in S2 cells. Cells were transiently transfected and induced to express each MMP for 48 h. Cell lysates were subject to reducing SDS-PAGE, transferred to nitrocellulose, and probed simultaneously with a rabbit polyclonal anti-FLAG antibody and a cocktail of three mouse monoclonal antibodies against the catalytic domain (anti-Mmp1catD) (panel A) or with a rabbit polyclonal anti-FLAG antibody and a mouse monoclonal anti-Mmp1Cterm antibody (panel B). Fluorescently-coupled species-specific secondary antibodies were imaged in separate channels. The merged image shows that bands are recognized by both the FLAG and Mmp1 antibodies. For loading controls, blots were stripped and reprobed for actin. In (**A**) signals for wild-type and E225A mutants (left and right) demonstrate that anti-Mmp1catD recognize both wild-type and mutant catalytic domains. In (**B**) the lack of signal for Mmp1-PJ demonstrates that anti-Mmp1Cterm antibodies recognize isoforms PF and PC but do not recognize PJ. (**C**) Comparison of the amino acid sequences of Mmp1-PF, PJ, and PC. The purple box highlights the region deduced to contain the anti-Mmp1Cterm epitope. (**D**) Western blots of Mmp2 and its catalytically inactive mutant (E258A) expressed in S2 cells. The E258A mutant reproducibly gave elevated levels of the ~90 kDa band compared to wild-type. Mmp2 expression and blotting were the same as for Mmp1. Full blot images are provided in Supplementary Information.

Despite their names, DmMmp1 and DmMmp2 are not orthologous to mammalian MMP-1 and MMP-2, and commercial antibodies against these vertebrate MMPs do not recognize the fly proteins. We previously reported generating mouse monoclonal antibodies against the recombinant catalytic domain of Mmp1^18^,^19^, referred to here as anti-Mmp1catD. To verify expression of our four tagged full-length MMP proteins, we expressed inducible constructs in S2 cells, and used anti-FLAG and anti-Mmp1catD on Western blots of cell lysates. As expected, the anti-FLAG antibodies recognized bands corresponding to all the tagged MMPs (Fig. 2). For Mmp1, generally the same bands were detected with the anti-Mmp1catD antibodies (Fig. 2A,B). Previous genetic studies have utilized catalytically inactive Drosophila MMP mutants *Mmp1*^E225A^ and *Mmp2*^E258A^ 22,25,27,28,29,30. We expressed these inactive mutants in S2 cells, and they were reproducibly expressed at elevated levels compared to the wild-type proteases. We determined that the Mmp1^E225A^ mutants were recognized by the anti-Mmp1catD antibodies despite the mutation in the catalytic site (Fig. 2A, right panel), and they ran similarly to their wild-type cognates.

At the same time that we generated the anti-Mmp1catD monoclonal antibodies, we had generated a monoclonal antibody against the Mmp1 C-terminal domain (see Methods). The anti-Mmp1Cterm antibody gives more non-specific background staining than our anti-Mmp1catD antibodies, so we have not found it useful until now. On staining our western blots with anti-Mmp1Cterm, we were surprised that it recognized Mmp1-PF and Mmp1-PC but not Mmp1-PJ (Fig. 2B), for both wild-type and mutant forms. This antibody was raised and selected against a recombinant C-terminal portion of Mmp1-PF, from residue Ile299 to the end, a region that includes the hemopexin domain shared by all isoforms and the variable C-terminal region. Since anti-Mmp1Cterm is able to recognize Mmp1-PC as well as PF, we reasoned that the monoclonal epitope lies between amino acid 501–530 of Mmp1-PF and Mmp1-PC, as this region is absent in Mmp1-PJ (Fig. 2C). Thus the anti-Mmp1Cterm antibody can be used to partially distinguish splice forms.

For Mmp2, western blots probed with anti-FLAG showed similar migration of the wild-type and mutant proteins, although reproducibly the ratio of the band intensities was altered, which may indicate rapid cellular turnover caused by the elevated levels of the inactive protein (Fig. 2D). In sum, all four proteins were expressed well in cell lysates, with apparent molecular weights of 65 kDa for Mmp1-PF, 60 kDa for Mmp1-PJ, 75 kDa for Mmp1-PC, and 110 kDa for Mmp2.

### Both Mmp1 and Mmp2 can be membrane-tethered and secreted

**Figure 3:**
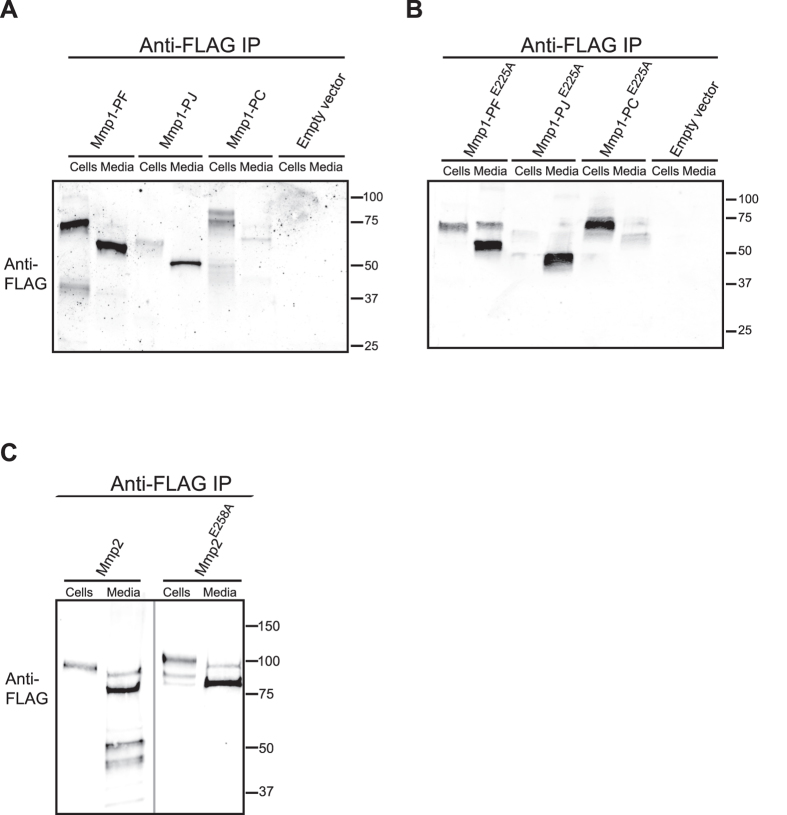
DmMmp1 and DmMmp2 are released from cells into the media. Each of the four full-length MMP isoforms were expressed in insect S2 cells for 48 h, immunoprecipitated from cell lysates or conditioned media with mouse anti-FLAG, and analyzed by western blotting with rabbit anti-FLAG as in Fig. 2. (**A**) Wild-type isoforms of Mmp1. Soluble Mmp1-PF was found in both cell lysates and media, soluble Mmp1-PJ was predominantly found in media, and GPI-anchored Mmp1-PC was predominantly found in cell lysates. The doublet of Mmp1-PC may represent auto-catalysis during immunoprecipitation, as it is not apparent in cell lysates (see Fig. 2A). (**B**) Inactive E225A mutants of Mmp1 isoforms behaved similarly to their wild-type cognates in panel A. (**C**) Mmp2 was precipitated from both cell lysates and from conditioned media, as was its E258A inactive mutant. The inactive mutant appeared more stable than wild-type after release into the media. The thin gray vertical line indicates that intervening lanes were omitted. Full blot images are provided in Supplementary Information.

To examine the differences between products of the two fly MMP genes, we asked whether they differentially remained associated with cells or were secreted into cell culture media. We immunoprecipitated each MMP from cell lysates or media with anti-FLAG, eluted with FLAG peptide, and analyzed the proteins by Western blot (Fig. 3). As expected, Mmp1-PF and Mmp1-PJ were both secreted into the media. Interestingly, each secreted band had an apparent molecular weight approximately 9 kDa smaller than its cellular counterpart (Fig. 3A); this is the size of the inhibitory pro-domain. The size change suggests that these enzymes are maintained as zymogens prior to secretion and are proteolytically activated coincident with secretion. Secretion-dependent activation is consistent with the predicted furin cleavage site RX[R/K]R found in both fly MMPs and ten mouse MMPs^31^. Expression of the catalytically inactive mutants of Mmp1-PF and Mmp1-PJ also results in their secretion into the media. In contrast to the wild-type MMPs, however, bands representing both zymogen and active protease were secreted, indicating inefficient removal of the pro-domain from the mutants (Fig. 3B).

Because they are predicted to have GPI-anchors, Mmp1-PC and Mmp2 were not expected to be precipitated from the media. Indeed, most Mmp1-PC was detected in cell lysates (Fig. 3A). The cell media contained a faint band of Mmp1-PC, with a smaller apparent molecular weight than the cell-associated Mmp1-PC, suggesting that further cleavage is associated with the soluble form. In contrast to Mmp1-PC, a significant amount of Mmp2 was detected in the media (Fig. 3C). In cell lysates a single band of Mmp2 is apparent, but in media multiple bands of FLAG-tagged Mmp2 were consistently apparent. These bands are found in the wild-type but not the inactive mutant, suggesting that the active Mmp2 is unstable in the media perhaps due to autocatalytic activity. In summary, contrary to previous descriptions, Mmp1 is found in both membrane-tethered and secreted forms, and Mmp2 is largely released into the media.

**Figure 4:**
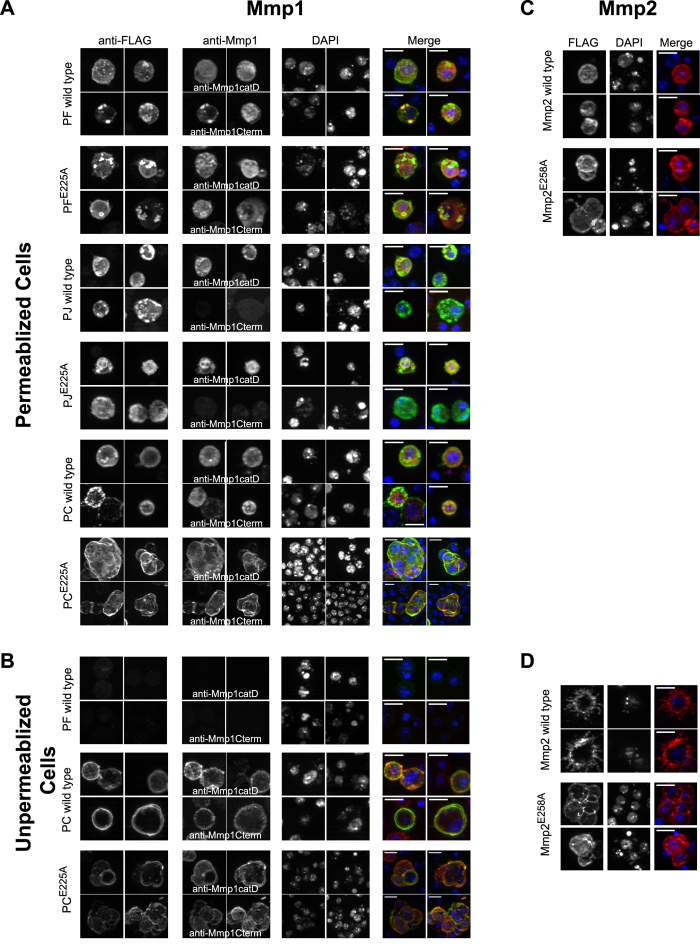
DmMmp1 and DmMmp2 are present on the external plasma membrane. Insect S2 cells were transfected with inducible Mmp1 and Mmp2 constructs and antibody-stained to determine protein localization. Rows are labeled on the left with the transfected protein and on top with the antibody. (**A**) Three Mmp1 isoforms, wild-type and inactive mutant, were transfected, induced, and co-stained with anti-FLAG, anti-Mmp1 (which recognizes endogenous as well as induced Mmp1 protein), and DAPI. Cells were permeabilized to reveal internal protein localization. Four images are shown for each construct. Because the Mmp1 catalytic and C-terminal monoclonals could not be used simultaneously (same species), the top two images in each row are stained with anti-Mmp1catD antibodies, and the bottom two images in each row are stained with anti-Mmp1Cterm antibodies. All three antibodies gave similar staining patterns. The exception was Mmp1-PJ, which was not recognized by the C-terminal antibody in cells or western blots. (**B**) Cells were treated as in A, except they were not permeabilized, to reveal protein located outside the plasma membrane only. Wild-type Mmp1-PF was not detectable without permeabilization. Also not detectable in unpermeabilized cells were Mmp1-PF^E225A^, Mmp1-PJ, and Mmp1-PJ^E225A^ (not shown). In contrast, Mmp1-PC and its inactive mutant localize to the plasma membrane. (**C**) Wild-type and inactive Mmp2 were transfected, induced, and stained with anti-FLAG in permeabilized conditions. Two cells are shown for each condition. (**D**) Cells were stained for Mmp2 as in (**C**) except that they were not permeabilized to reveal protein located outside the plasma membrane only. Mmp2 wild-type and mutant protein localizes to the plasma membrane, and Mmp2 wild-type protein decorates fine cytoplasmic extensions. All scale bars in (**A–D**) are 10 μm.

To assess whether MMPs are tethered to the cell surface, S2 cells overexpressing the four wild-type cDNAs and their inactive mutants were stained for MMP localization, with or without cell permeabilization (Fig. 4). For the six Mmp1 constructs, we co-stained cells with mixtures of either 1) anti- FLAG and anti-Mmp1catD antibodies, or 2) anti-FLAG and anti-Mmp1Cterm antibodies (Fig. 4A). As expected for proteins with signal peptides, we observed secreted Mmp1-PF and Mmp1-PJ (both wild-type and mutant) throughout permeabilized cells. This result is similar to the previously reported *in vivo* epidermal staining pattern^23^. As with the western blots, Mmp1-PJ was not recognized by anti-Mmp1Cterm but was recognized by both the anti-FLAG and anti-Mmp1catD antibodies. When the cells were not permeabilized, no signal was observed with any of the antibodies for Mmp1-PF, Mmp1-PJ, or their inactive mutants (Fig. 4B shows images for wild-type Mmp1-PF, others not shown). These data and our immunoprecipitation data support the accepted model that Mmp1-PF and Mmp1-PJ are not retained at the cell surface, but rather they are secreted and released from S2 cells.

The MMPs with predicted GPI-anchors, Mmp1-PC and Mmp2 and their mutants, were localized throughout permeabilized cells (Fig. 4A,C). Strikingly, the cell-surface signals were intensified in unpermeabilized cells, demonstrating that these proteins localize to the outside of the plasma membrane (Fig. 4B,D). These results support the prediction of GPI anchors for Mmp1-PC and Mmp2.

### Mmp1-PC and Mmp2 are tethered to the membrane by GPI anchors

**Figure 5:**
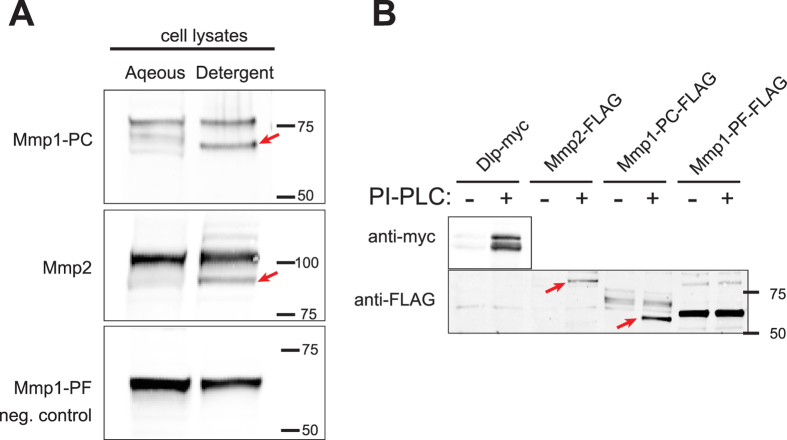
DmMmp1-PC and DmMmp2 have GPI anchors. (**A**) S2 cells induced to express tagged Drosophila MMPs were lysed, partitioned by TritonX-114 into aqueous and detergent phases, and analyzed by anti-FLAG western blot. Bands of Mmp1-PC and Mmp2 partitioned preferentially to the detergent phase as hydrophobic proteins (red arrows), consistent with a lipid modification such as glypiation. Mmp1-PF is a negative control that shares 91% identity with Mmp1-PC, and it partitioned similarly to both phases. (**B**) Cells were treated or not with the GPI-cleaving enzyme PI-PLC, and the proteins released by GPI-cleavage were analyzed by Western blot. GPI-cleavage released unique forms of Mmp1-PC and Mmp2 (red arrows), as it did for the GPI-anchored protein Dlp-myc (positive control). In contrast, secreted Mmp1-PF (negative control) was released from cells with or without PI-PLC treatment. Full blot images are provided in Supplementary Information.

Both Mmp1-PC and Mmp2 are predicted to have their C-termini replaced with GPI anchors according to Pred-GPI^32^, but this post-translational lipid modification has not been experimentally confirmed. TX-114 two-phase partitioning can be used to identify hydrophobic proteins, including those with lipid moieties, which are enriched in the detergent-enriched compared to the detergent-depleted (aqueous) fraction^33^. On partitioning cell lysates, we found a band of Mmp1-PC highly enriched in the detergent fraction (arrow, Fig. 5A), whereas the other Mmp1-PC band did not display preferential partitioning and likely represents the immature protein before glypiation. Similarly, a band of Mmp2 preferentially partitioned to the detergent phase (arrow, Fig. 5A). As a control, Mmp1-PF expressing lysates were also tested, as Mmp1-PF is not predicted to have a GPI anchor but shares 91% amino acid identity with Mmp1-PC. The single band of Mmp1-PF partitioned to both phases, like the immature bands of Mmp1-PC and Mmp2. These results demonstrate that Mmp1-PC and Mmp2 can partition like lipid-modified proteins, whereas Mmp1-PF cannot.

In order to determine the molecular identity of the modification responsible for this partitioning behavior, we treated intact cells with PI-PLC, which cleaves GPI anchors specifically. This assay has been used previously to identify GPI anchors on MMPs^34^,^35^. A positive control, the GPI-linked protein Dlp, was released from cells into the buffer following PI-PLC treatment (Fig. 5B). PI-PLC treatment released a unique smaller band of Mmp1-PC and a single species of Mmp2 (Fig. 5B, arrows). A negative control, Mmp1-PF, was released into the buffer regardless of whether the cells were treated with PI-PLC. Moreover, PI-PLC treatment did not change the mobility of Mmp1-PF in the SDS-PAGE gel. Despite our efforts, we were unable to determine if removing the GPI anchor from the MMPs with PI-PLC altered their TX-114 partitioning, because the proteases were unstable (proteolyzed) during phospholipase treatment. Taken together, these three assays – the localization of Mmp1-PC and Mmp2 on the cell surface, their partitioning to TX-114 detergent layer, and their release by PI-PLC – demonstrate that these MMPs are modified to have GPI anchors, which tether these MMPs to the cell surface.

## Inactive GPI-linked MMPs promote cell adhesion

**Figure 6:**
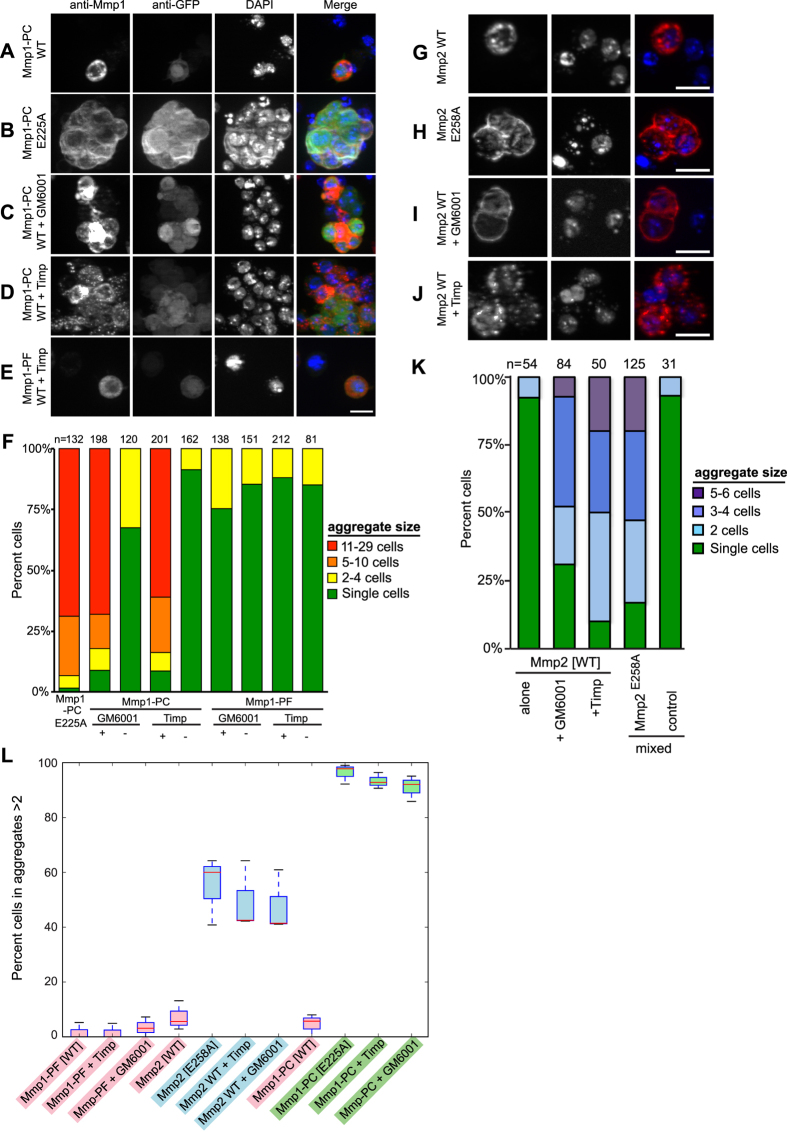
GPI-anchored Mmp1, and to a lesser extent Mmp2, promote homotypic cell aggregation when catalytic activity is inhibited. S2 cells were transiently transfected as indicated, induced for 48 h, resuspended and allowed to settle for 1 h, then fixed, permeabilized, and stained. (**A**) Cells expressing wild-type Mmp1-PC were identified most often as single cells. (**B**) Cells expressing catalytically inactive Mmp1-PC^E225A^ aggregated in large clumps. (**C,D**) Aggregation was observed when cells expressing wild-type Mmp1-PC were treated continuously with the catalytic inhibitor GM6001 from the start of induction (**C**) or when cells were co-transfected with wild-type Mmp1-PC and DmTimp (**D**). (**E**) Cells co-transfected with secreted Mmp1-PF and DmTimp do not aggregate, as this phenomenon is specific to membrane-anchored Mmp1-PC. (**F**) Quantification of aggregation caused by expression of inactive Mmp1. Cells expressing Mmp1-PC do not aggregate when catalytically active (columns 3 and 5), and cells expressing soluble Mmp1-PF do not aggregate under any circumstances (columns 6–9). (**G**) Cells expressing wild-type Mmp2 do not aggregate. (**H**) Cells expressing catalytically inactive Mmp2^E258A^ have a low level of aggregation. (**I,J**) Similar low levels of aggregation were observed in cells expressing wild-type Mmp2 treated continuously with GM6001 (**I**) or co-transfected with DmTimp (**J**). (**K**) Quantification of aggregation caused by expression of inactive Mmp2. Note that many fewer cells aggregate in each clump than for Mmp1-PC. (**L**) Comparison of aggregation frequency in Mmp1-PF, Mmp2, and Mmp1-PC expressing cells. Cells were considered to be aggregating if they were in clusters containing more than two cells. Over 100 cells were counted for each condition, and all experiments were performed in triplicate. The red line marks the median, and whiskers mark the range of the data. Color (pink, blue, green) indicates significance value, i.e. all pairwise comparisons between different color groups were highly significant with P values less than 0.0002, whereas all pairwise comparisons within a color group were not significantly different with P values greater than 0.9. Statistical analysis methods were ANOVA with post-hoc analysis with both Tukey HSD and with Bonferroni corrected pairwise t-tests. All scale bars are 10 μm. Scale bar for (**A–E**) is shown in panel E.

Surprisingly, cells expressing inactive Mmp1-PC^E225A^ consistently showed a dramatic and unexpected change in morphology. They aggregated tightly to each other, often in groups of about 10–25 cells (Fig. 4A last row and Fig. 6B,F,L), a phenotype not observed with any other Mmp1 isoform (Figs 4A and 6A,F,L). We confirmed that cells aggregated, rather than fused, by staining with CellMask, which clearly identified cell membranes (data not shown, see Methods). Because E225A is a mutant form of Mmp1-PC not found in nature, the significance of this result was not immediately clear. To determine if pharmacological inhibition of Mmp1-PC was sufficient to aggregate cells, transiently-transfected catalytically active Mmp1-PC expressing cells were treated with the broad spectrum MMP catalytic inhibitor GM6001. Mmp1-PF-expressing cells were used as a negative control. Aggregation of cells transfected with Mmp1-PC, but not Mmp1-PF, was observed in the presence, but not the absence, of GM6001 (Fig. 6C,F,L). DmTimp is an endogenous inhibitor of Mmp1^18^,^36^. To determine if Timp can also induce aggregation, we co-transfected cells with wild-type Mmp1-PC and Timp. Aggregation was observed at levels comparable to the other inhibited Mmp1-PC conditions (Fig. 6D,F,L). In contrast, aggregation was never observed when cells were transfected with any of the other Mmp1 catalytically inactive mutants (Fig. 4) nor when Mmp1-PF cells were treated with GM6001 or co-transfected with Timp (Fig. 6E,F,L). Because Timp and Mmp1-PC may be co-expressed in cells *in vivo*, these data suggest that Timp may convert Mmp1-PC into a promoter of cell aggregation or adhesion in tissues.

After investigating aggregation in cells overexpressing Mmp1-PC, we looked carefully at cells overexpressing the other GPI-anchored fly MMP, Mmp2. Although more subtle, aggregation was evident when cells expressed an inactive form of Mmp2. The E258A mutation, GM6001 treatment, or co-expression with Timp each resulted in aggregation of expressing cells (Fig. 6G–L). Cells were plated at the same density for all aggregation experiments. To rigorously test if subtle differences in growth rates could underlie the aggregation rates by altering cell density, we transformed separate batches of cells with Mmp2^E225A^ or GFP, and then mixed them together. We found that only the Mmp2^E225A^ transformed cells, but not the GFP transformed cells mixed in with them, were able to aggregate (Fig. 6K, last column). This result also indicates that cell aggregation is a cell-autonomous phenotype. In another analysis, aggregation levels in MMP-expressing cells were compared as the percent of cells found in aggregates composed of more than 2 cells, because mitosis can result in two-cell aggregates even in non-aggregating cells. This analysis (Fig. 2L) identified three distinct aggregation phenotypes: one class did not aggregate (shown in pink), and this class included wild-type MMPs and inhibited Mmp1-PF. Another class had moderate aggregation (shown in blue) with about half the cells found in aggregates of three or more, and this class included the three inhibited conditions of Mmp2. The third class had extreme aggregation (shown in green), with nearly all cells found in aggregates of three or more cells, and this class included the three inhibited conditions for Mmp1-PC. The classes were established by ANOVA, as members of different classes were highly signifcantly different from each other, but within classes there were no significant differences (see Methods). Thus both fly GPI-anchored MMPs, Mmp1-PC and Mmp2, mediate S2 cell adhesion when they are rendered inactive.

## Mmp1 and Mmp2 cleave different substrates

*In vivo*, it has been unclear if the differences between *DmMmp1* and *DmMmp2* represent differences in substrate cleavage or differences in expression and subcellular localization. Biochemical analysis of recombinant catalytic domains found that both fly MMPs cleave the same synthetic fluorogenic substrates (Q24,Q41, and Knight)^16^,^17^,^36^. Additionally, Mmp1 was found to cleave collagen IV, fibronectin, and casein from vertebrates^16^. However, since no similar substrate analysis has been performed for DmMmp2, it has been unclear if it has a similar substrate profile to DmMmp1. The substrates of the fly proteases cannot be inferred from mammalian studies, because there is no clear correspondence between the fly and mammalian MMPs.

**Figure 7:**
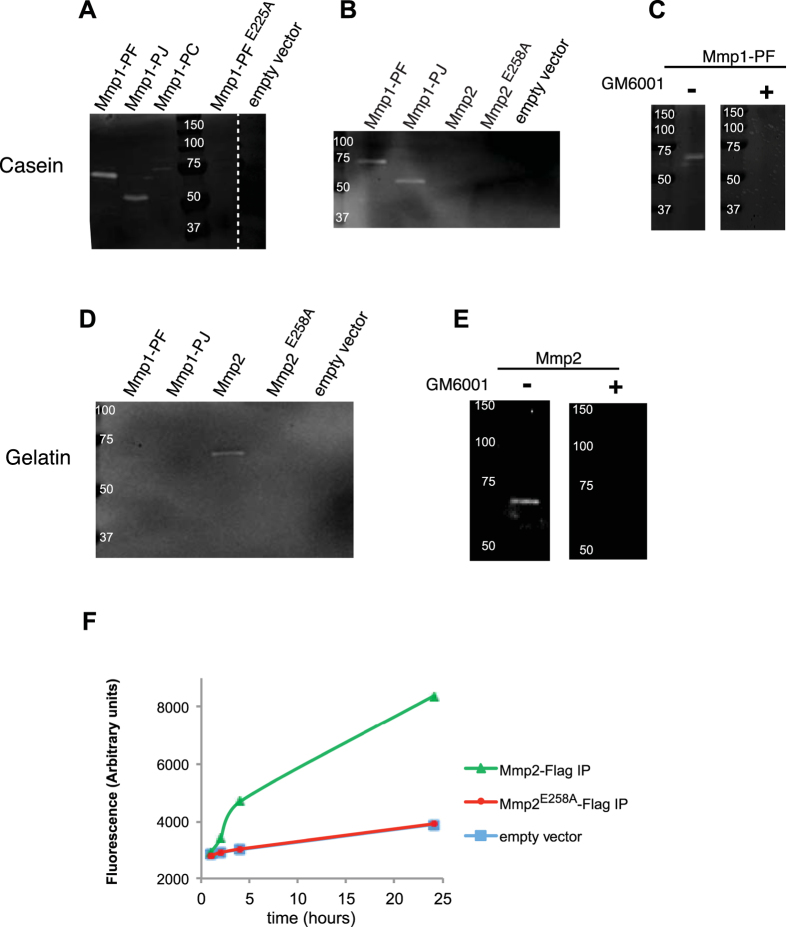
DmMmp1 and DmMmp2 cleave different substrates. Cells were transiently transfected with FLAG-tagged MMPs and induced for 48 h. MMPs were immunoprecipitated from conditioned media, eluted with FLAG peptide, concentrated, and analyzed for MMP proteolytic activity by gelatin or casein zymography **(A–E)** or with fluorogenic gelatin **(F)**. (**A**) All Mmp1 isoforms are active on casein zymograms, although GPI-anchored Mmp1-PC is present at much lower levels in media than the soluble isoforms. As predicted, the Mmp1^E225A^ mutant is not active. (**B**) In contrast to Mmp1, Mmp2 has no detectable activity on casein. (**C**) An immunoprecipitate of Mmp1-PF was divided and treated or not with GM6001, and subjected to casein zymography, in parallel. GM6001 effectively inhibits DmMmp1 activity. (**D**) On a gelatin zymogram, DmMmp1 has no detectable activity but DmMmp2 does. As predicted, the Mmp2^E258A^ mutant is not active. (**E**) An immunoprecipitate of Mmp2 was divided and treated or not with GM6001, and subjected to gelatin zymography, in parallel. GM6001 effectively inhibits DmMmp2 activity. (**F**) Mmp2, but not Mmp2^E258A^, cleaves the fluorogenic substrate DQ-gelatin. DmMmp2-FLAG was immunoprecipitated from media conditioned by transiently transfected S2R+ cells. Full zymogram gel images are provided in Supplementary Information.

We tested the enzymatic activity of immunoprecipitates of full-length fly MMPs from cells and media by casein and gelatin zymography. For zymography, the sample containing protease is denatured in SDS and electrophoresed through a gel containing a potential protein substrate (here casein or gelatin); the SDS is washed out and the enzyme partially refolds; and finally the enzyme cleaves the in-gel substrate during a subsequent incubation. Active enzyme is recognized by loss of the in-gel substrate visualized by Coomassie staining, leaving a clear band on a blue background^37^. Using conditioned media as the MMP source, zymography shows that Mmp1-PF, PJ, and PC all cleave casein (Fig. 7A), consistent with their sharing the same catalytic and hemopexin domains. The zymography band for Mmp1-PC was very faint because this isoform is mainly held at the cell membrane (Fig. 3A). Thus all FLAG-tagged Mmp1 full-length proteins retain catalytic activity after immunoprecipitation. We tested the effectiveness of the E225A mutation, and as expected this mutation abolishes the catalytic activity of Mmp1 on casein (Fig. 7A). No zymography substrate has been reported for Mmp2, and we found that full-length Mmp2 did not cleave casein (Fig. 7B).

In contrast, zymography showed that Mmp2 cleaved gelatin, whereas Mmp1 was unable to cleave gelatin (Fig. 7D). Mmp2 was immunoprecipitated from the media, where the highest levels of Mmp2 protein were detected. Further, we used zymography to analyze the Mmp2^E258A^ mutant that was designed to be catalytically dead. Mmp2 mutants had substantially reduced activity, though not eliminated, on gelatin gels (below detection threshold in Fig. 7D). We rejected the hypothesis that induced Mmp2^E258A^ dimerizes with wild-type endogenous Mmp2 by co-expressing wild-type and mutant Mmp2 with different tags, to ascertain whether the mutant could pull down the wild-type, and it did not (data not shown). We note that residual MMP catalytic activity on zymograms has been reported previously for similar E-to-A mutants^38^. To evaluate Mmp2 activity in a different assay, we used a commercially available fluorogenic gelatin substrate, DQ-gelatin, and evaluated the Mmp2-dependent generation of a fluorescent signal. We found that Mmp2 immunoprecipitated from the media was able to cleave the substrate, but the E258A mutant had no activity and was indistinguishable from the control IP performed on cells transformed with an empty vector (Fig. 7F). Thus we conclude that the E258A mutant is inactive, as predicted. The different activities with different zymography assays establishes unequivocally that the two fly MMPs have different substrate specificities.

The active site is highly conserved between flies and mammals, as demonstrated by cross-species inhibition: DmMmp1 is inhibited by DmTimp and also by mammalian TIMP-2 and TIMP-4, and reciprocally DmTimp inhibits human MMP-1, -2, -3, -14, and ADAM-10 and ADAM-17^16^,^36^. Because of this conservation, we tested the broad-spectrum MMP inhibitor GM6001 as an inhibitor in parallel samples that were treated or not with the inhibitor. GM6001 completely inhibited Mmp1 and Mmp2 catalytic activity on zymograms (Fig. 7C,E), which makes it a useful tool for future studies.

## Discussion

With only two MMP genes, Drosophila has the fewest MMPs of any known animal model, as *C. elegans* has six MMPs, mice have 23, zebrafish have 25^26^,^39^. The simplicity of fly MMPs offers an unparalleled opportunity to understand the nature of redundancy, specificity, and diversity in the MMP family. What is the essential difference between these MMPs that the fly requires two of them? Previous studies have described the two Drosophila MMPs as having distinct pericellular localization, with Mmp1 secreted and Mmp2 membrane-anchored^16^,^17^,^18^,^23^,^26^. These pericellular localization differences might imply that the fundamental distinction in the MMP family is whether or not an MMP is membrane associated. This distinction would emphasize the classifications of the 7 vertebrate TM-MMPs versus 16 secreted MMPs. In this study, however, we find that both Mmp1 and Mmp2 can be secreted or remain membrane associated. Specifically, we find that in addition to the expected soluble Mmp1 splice isoforms secreted from cells (Mmp1-PF and -PJ), the Mmp1-PC splice isoform has a GPI anchor tethering it to the cell membrane, as evidenced by its increased hydrophobicity, its localization at the outer cell surface, and its release from cells by PI-PLC. The tissues and developmental stages that express Mmp1-PC are presently unknown. Such information is difficult to determine from RNA-seq data^40^,^41^ because exon 9, which encodes the GPI-anchor sequence, is included as 3′ UTR in several Mmp1 transcripts (Fig. 1).

We demonstrate that Mmp2 has a GPI anchor, as the protein is shed from the cell in response to PI-PLC, it has high hydrophobicity, and it localizes to the outside of the plasma membrane. Unexpectedly, however, we find Mmp2 accumulates in the media. We suspect that after Mmp2 reaches the cell surface, the GPI anchor is cleaved, releasing the protease from the cell. Mmp2 found in the media has activity by zymography. Similar constitutive release has been observed for human MT6-MMP/MMP-25, also a GPI-anchored MMP^42^,^43^. Regardless of mechanism, our results that active Mmp2 is found abundantly in the media imply that Mmp2 can also be released from cells to act at a distance *in vivo*.

Since both Mmp1 and Mmp2 are both membrane bound and released from cells, what is the important difference between them? We propose that the critical difference between the two enzymes is their distinct substrate specificities, and this difference requires the fly to maintain both genes. This difference was not evident from previous studies, which found that recombinant catalytic domains of each fly MMP cleave the same three fluorogenic substrates^16^,^17^. The substrate specificity we observe might be related to our use of full-length proteases that retain important binding sites in addition to the catalytic domain. Although neither of the zymography substrates we utilized, casein or gelatin, is endogenous to fly, the specificity of each MMP toward them indicates that these proteases have different substrate repertoires. Different repertoires are also suggested by the fact that, of the few endogenous substrates identified to date, none of them are shared. For Mmp1, the substrates Ninjurin A, E-Cadherin, and Pigment Dispersing Factor have been identified^20^,^25^,^27^; and for Mmp2, a fibrillin-like molecule Frac, collagen IV, and the glypican Dally-like protein (Dlp) have been identified^2^,^24^,^44^,^45^. These different substrates also explain why they are not genetically redundant, as each MMP mutant has a lethal phenotype. Interestingly, recent studies comparing mammalian MMP substrate specificities in diverse substrate space have found subtle differences among the specificites of this much larger family^46^,^47^,^48^. These findings, in concert with ours, support the idea that substrate specificity is the key distinction between different MMP family members.

We hope that some of our methods and findings reported here will assist in the future biochemical analysis of MMPs and their substrates in flies. We generated tagged stable constructs of four full-length MMP isoforms and demonstrated their activity. We determined the specificity of the Mmp1 monoclonal antibodies, which are publicly available at the Developmental Studies Hybridoma Bank, and found, first, that the anti-Mmp1Cterm antibody does not recognize Mmp1-PJ, and second, that the anti-Mmp1catD antibodies recognize the catalytically dead as well as the wild-type proteins. This work also identified fluorogenic and zymography assays for Mmp2 activity, and we used these and a previously reported zymography assay for Mmp1 activity to test the assumption that the catalytically inactive mutants (which are widely used *in vivo*) indeed do not have catalytic activity, and to demonstrate the effectiveness of GM6001, a useful pharmacological inhibitor of fly MMPs.

Finally, we identified a novel and surprising function specifically associated with the inactive GPI-anchored MMPs. Inactive Mmp1-PC can mediate tight cell-cell adhesion when the catalytic domain is mutated, inhibited by GM6001, or inhibited by Timp, and a less obvious but similar phenotype was observed for inactive Mmp2. Considering the widespread expression of Timp, Mmp2, and the Mmp1 glypiation sequence by RNAseq data, it is likely that GPI-anchored MMPs and Timp are co-expressed in many tissues, where they may promote this novel MMP-mediated function. Neither GPI-anchored MMPs have the potential to mediate cell-cell adhesion directly because the GPI anchor has no way to interact with the cytoskeleton. Thus an inactive protease must interact with a binding partner in *cis*, and this interaction indirectly results in a change in cell adhesion. Why only inactive and not active GPI-anchored proteases can mediate cell adhesion is unclear. It is known that MMPs can have important non-catalytic functions. For example, the MMP-3 hemopexin domain and the MMP-14 transmembrane/cytoplasmic domain are critical for branching and invasion of mammary gland cultures^49^,^50^. Additionally, the hemopexin domains of MMP-12 and MMP-9 are required for antimicrobial function and for migration, respectively^51^,^52^. Similarly, we have reported that the DmMmp1 hemopexin domain is important for developmental tissue invasion during metamorphosis *in vivo*^28^. Further, catalytically inactive GPI-anchored MMP-17 and the EGFR cell-surface protein indirectly cause cell proliferation. Despite these precedents, we are unaware of another case of a function ascribed solely to an inactive MMP that is not shared by its active cognate. Our aggregation data suggest a new function for GPI-anchored MMPs.

## Methods

### Molecular biology and plasmids

Plasmids used to induce gene expression were variants of *pRmHa3*, which carries the copper-inducible metallothionine promoter. Plasmids used to drive wild-type MMP expression were *pRmHa3_C-FL2-Mmp1.f1* (encoding Mmp1-PF), *pRmHa3_C-FL2-Mmp1.f2* (encoding Mmp1-PJ), *pRmHa3-Mmp1-PC-E548-3xFL, pRmHa3-Mmp2-S710-3xFL*, all of which were constructed for this study by standard methods. cDNAs encoding Mmp1-PF, Mmp1-PJ and Mmp2 were identified in ref. 18, and the cDNA encoding Mmp1-PC was obtained from the Drosophila Genomic Resources Center (Bloomington, IN). Inactive MMPs were expressed from derivative plasmids harboring mutation E225A for Mmp1 and E258A for Mmp2. *pRmHa3_C-FL2* was used as the empty vector control, and *pRmHa3-GFP* and was used as a transfection control in Fig. 6K. DmTimp was expressed with the *pUAST-Timp*^18^ and the positive control Dlp was expressed with *pUAST-Dlp-myc*^2^, each co-transfected with *pRmHa3-Gal4*.

To generate FLAG-tagged Mmp2, five positions within Mmp2 were tested for insertion of a large epitope, GFP. The five positions were 1) at the N-terminus just after the pro-domain, inserted after R136, 2) in the catalytic domain, inserted after W151, to model the function of Drosophila lines with GFP recombined into MiMIC lines MI00489, MI03429, or MI02914^21^, 3) in the hinge domain, inserted after R477, 4) just before the GPI anchor, inserted after S710 and 5) at the C-terminus, inserted after S734. All the Mmp2-GFP constructs were secreted into the media except for the insertion in the catalytic domain, which was found in the cell lysates only. This results indicates that the GFP tag recombined into the MiMIC lines would interfere with Mmp2 protein secretion *in vivo*. We excluded the catalytic domain fusion from further consideration The N-terminal fusion was eliminated because the size of the protein in the media indicated internal cleavage. The C-terminal fusion was excluded because it was not glypiated, evidenced by retention of the epitope. Both the hinge fusion and the pre-GPI fusion appeared as doublets in the cell lysates, suggesting normal processing of the zymogen to the active enzyme, but the hinge fusion exhibited a unique degradation product at low levels. We transformed a construct encoding the pre-GPI fusion protein into Drosophila and determined that it was functional, as we reported elsewhere^2^. We then replaced this GFP moiety with the smaller FLAG epitope.

To generate FLAG-tagged Mmp1-PC, three positions were tested for the FLAG epitope. The protein was unstable when FLAG was placed at the C-terminus, likely because we interfered with glypiation at the C-terminus. Similarly, when FLAG was placed after residue 134 at the N-terminus of the catalytic domain immediately after the pro-domain, the protein was not catalytically active in zymography. We generated stable and active enzyme by inserting a 3XFLAG epitope directly after E548, near the C-terminus before the GPI anchor sequence (Fig. 1C), and we generated an inactive mutant Mmp1-PC^E225A^ with the same tag.

### Cell culture and transfection

Drosophila S2 cells were maintained at 25° and S2R+ cells at 27° (in Fig. 7F) in Schneider’s medium (Gibco) supplemented with 10% heat inactivated Fetal Bovine Serum (Biowest) and 50 U/ml Pen-Strep (Gibco). Plasmids were transiently transfected by standard CaCl_2_ methods, resulting in transient transfection of ~20–30% of cells. Briefly, 6 μg total plasmid DNA was resuspended in 250 μl 240 μM CaCl_2_, and added over 2 min. with agitation dropwise to an equal volume of 2X HEPES Buffered Saline (Research Products International, H17100). After incubation for 30 min. at RT, this DNA mixture was added dropwise to one well of a 9 cm^2^ plate seeded the previous day with 1.5 × 10^6^ cells containing 3 ml media. After 16 h, the transfection media was replaced with fresh media. Gene expression was induced from the metallothionine-promoter by the addition of 500 μM CuSO_4_ to the media, starting 16 h after transfection for a total induction period of 48 h.

### Anti-Mmp1Cterm antibody

Hybridoma line 14A3D2 (monoclonal antibody) was raised against the C-terminal portion of Mmp1-PF, amino acids 299–541. Specifically, antibodies were first selected with a recombinant N-terminal GST-fusion of amino acids 299–541, and then screened with an N-terminal MBP-fusion of the same region, resulting in the identification of line 14A3D2. Although this study is the first report of 14A3D2, it has been available from the DHSB since 2006. This antibody was expected to be specific for the hemopexin domain, which is contained in the antigenic region 299–541, and accordingly the DSHB originally referred to the 14A3D2 antigen as the Mmp1 hemopexin domain. However, studies here indicate that the epitope lies further C-terminal, in an alternatively spliced region (Fig. 2). Thus, we refer to the 14A3D2 antibody as anti-Mmp1Cterm. We obtained 14A3D2 supernatant from the DHSB, and used it at 1:100 for WB or 1:50 for IF.

### Immunoprecipitation, western blotting, cell staining

Other primary antibodies used were rabbit anti-FLAG (Sigma F7425, 1:2000 for WB or 1:500 for IF), a cocktail of three anti-Mmp1 monoclonal antibodies raised against the catalytic domain, called anti-Mmp1catD (3A6B4, 3B8D12, and 5H7B11 maintained by the DSHB, 1:100 for WB or 1:50 for IF), mouse anti-GFP (Roche 11814460001, 1:1000 for WB), mouse anti-actin clone C4 (Millipore MAB1501R, 1:1000 for WB), and mouse anti-myc (Invitrogen 46–0603, 1:2000 for WB).

For immunoprecipitation from cell lysates, one well of cells (~2–4 × 10^6^ cells) was transiently transfected to express FLAG-tagged MMPs overnight, induced for 48 h, pelleted, and lysed with 500 μl Lysis Buffer (50 mM Tris HCl, pH 7.4, with 150 mM NaCl and 1% Triton X-100 and Halt protease inhibitors (Thermo-Fisher)). Lysate was applied to 100 μl resuspended anti-FLAG M2 resin (Sigma, mouse monoclonal) overnight at 4 °C, and protein was eluted with 250 μl 3X FLAG peptide (Sigma, 5 μg/μl in TBS). For subsequent blotting, 20 μl was run per lane, equivalent to precipitate from approximately 2 × 10^6^ cells. For immunoprecipitation from media, 3 ml of media was conditioned by cells for 2 days as above, and cells and debris were removed from 1 ml of media by spinning at 15,700 RCF for 15 min. 0.1% Tween 20 and protease inhibitors were added to media, which was applied to 100 μl resuspended resin and eluted with 250 μl FLAG peptide as above. For subsequent blotting, 20 μl was run per lane, equivalent to media conditioned by approximately 8 × 10^5^ cells for two days. For blots of whole cell lysates, lysate equivalent to 4 × 10^6^ cells was loaded per lane.

For Western blotting, samples were run on 10% SDS PAGE gels, transferred to nitrocellulose (GE Healthcare), and incubated with 1° antibody overnight at 4°, followed by 2° antibody incubation (Li-Cor) at 1:8000 for 1 h at RT. Blots were developed and imaged with the Odyssey Infrared Imaging System (LI-COR Biosciences). Blots in Fig. 2A,B were probed simultaneously with rabbit anti-FLAG and mouse anti-Mmp1catD or anti-Mmp1Cterm. Specificity of secondary antibodies can be observed by the lack of an anti-Mmp1Cterm band in the Mmp1-PJ lanes. After imaging MMPs, blots in Fig. 2 were stripped and reprobed for actin to assess gel loading. Membranes were stripped in NewBlot Nitro Stripping Buffer (Li-Cor 928–40030) twice for 15 minutes each with gentle agitation, and rinsed in 1XPBS.

For cell staining, cells were fixed on 10-well multitest slides (MP Biomedicals) with 2% EM-grade paraformaldehyde (Ted Pella), permeabilized with 0.1% Triton X-100 in PBS for 15 minutes, blocked with 1% NGS and 1% BSA, and stained with 1° antibodies 1 h at RT. For non-permeabilized cells, Triton X-100 was omitted from all steps and the paraformaldehyde was freshly opened. Secondary antibodies (Jackson) were Cy3 Donkey Anti-Mouse (715-165-150), FITC Donkey Anti-Rabbit (711-095-152), Cy3 Donkey Anti-Rabbit (711-165-152) and Cy5 Donkey Anti-Rabbit (711-175-152), used at 1:200. To visualize membranes, cells were stained with CellMask (Life Technologies C10045) at 5 μg/ml for 5 min before fixation^53^. Cells were mounted in Vectashield (Vector) containing DAPI. Images were collected with a Zeiss M2 epifluorescence microscope fitted with an Apotome for optical sectioning, and max-projections of Z-stacks are displayed. For visualization of protein localization, exposure times were optimized for individual panels. Thus, protein levels can be compared in western blots in Figs 2 and 3 but not from images in Figs 4 and 6.

### Counting cell aggregates

To quantify the percent cells found in aggregates of varying sizes, we examined fields of MMP-FLAG expressing cells that had been fixed, permeabilized, and stained for FLAG and DAPI, sometimes with a GFP transfection control (in Fig. 6A–E). We identified random fields of cells with the 20X objective and analyzed every MMP-FLAG-expressing cell in the field. To be counted as aggregated, rather than simply near to each other, cells had share an adjoining edge. Cells were counted such that a single aggregate containing 5 cells was counted as 5 cells within the relevant bin (e.g., “5–10 cells”). For the cell mixing control (Fig. 6K), equal numbers of S2 cells were separately transformed with *pRmHa3-Mmp2E258A-S710-3xFL* or the GFP control plasmid *pRmHa3-GFP.* After transfection reagents were removed, cells were replated together in one well and induced together with CuSO_4_. For the comparison in 6 L, cell aggregation was compared using two bins, non-aggregating (single cells or two-cell aggregates) vs. three or more cells in an aggregate. Greater than 100 cells were counted for each condition, and cells in aggregates were represented as a fraction of the total cells counted. All experiments were performed in triplicate. The percent cells in aggregates was plotted in matplotlib version1.5.3^54^ as a boxplot. ANOVA was performed in RStudio v. 1.0.136; R version 3.3.2^55^. There was a significant effect of MMP activity on the percent cells in aggregates at the p < 0.001 level [F(10,22) = 91.2, p = 1.55e-15]. Post-hoc analysis was conducted with both Tukey HSD and with Bonferroni corrected pairwise t-tests.

### Activity assays

For zymography, proteases were eluted from FLAG-M2 resin exactly as described for immunoprecipitation. 20 μl of protease-containing elute was resuspended in 2X Novex non-reducing sample buffer (Invitrogen) for all gels and all samples. Samples were run on Novex Zymogram Gels, either 10% gel containing 0.1% gelatin (Invitrogen EC6175BOX) or 12% gel containing 0.05% casein (Invitrogen EC6405BOX). After electrophoresis, gels were incubated with Novex Renaturing Buffer and Novex Developing Buffer, then stained with Colloidal Blue Kit, all according to manufacturer’s instructions (Invitrogen). When used, inhibitor GM6001 (Santa Cruz) was added to cell culture media at a final concentration of 25 μM at the time of induction with the CuS0_4_. Additionally, GM6001 was added to protein samples at 25 μM before electrophoresis, and to Renaturing and Developing buffers at 10 μM. DMSO vehicle alone was added to GM6001 control samples.

The Mmp2 fluorogenic activity assay utilized DQ gelatin from pig skin, labeled with fluorescein, as part of the Enz-Chek Gelatinase/Collagenase Assay Kit (Invitrogen E-12055), following manufacturers’ instructions except using 20 μg/ml substrate. Mmp2 was immunoprecipitated onto M2 FLAG resin from media conditioned for 48 h by transiently transfected S2R+ cells. Resin-bound immunoprecipitate was added directly to the substrate and incubated at 37 °C. After different time points, resin was spun down, and the fluorescence intensity of the supernatant was measured on BIO-TEK synergy HT microtiter plate reader at 485/20 nm excitation and 528/20 nm emission.

### GPI analysis

TX-114 partitioning was performed by lysing 2 × 10^6^ transiently transfected cells in 500 μl cold TBS with protease inhibitors to which was added 100 μl precondensed TX-114 (~12% detergent) in TBS^56^. Cells were lysed for 15 minutes on ice with gentle mixing. Insoluble material was removed from the lysate by spinning at 4 °C. Cleared lysates were warmed to 37° for 3 minutes and phase partitioning was accomplished by spinning at 1000Xg for 10 min at RT. Upper (aqueous) and lower (detergent) phases were collected separately. Detergent phase was diluted five-fold with TBS and 20 μl of each phase was subject to SDS-PAGE and western blotting.

PI-PLC cleavage was performed by resuspending ~10^6^ transiently transfected S2 cells in 400 μl PBS with 0.5 U PI-PLC (Life Technologies Corp) or vehicle control with gentle agitation for 20 min at 4 °C. Cells were pelleted and 40 μl supernatant was mixed with sample buffer and subject to SDS-PAGE and western blotting. PI-PLC effectiveness was verified by staining non-permeabilized cells for FLAG after treatment (data not shown). Cells were rinsed quickly in PBS and allowed to settle for 1 h in media onto 10-well multitest slides. Cells were fixed with fresh 2% EM-grade paraformaldehyde (Ted Pella) then blocked and stained as described.

## Additional Information

**How to cite this article:** LaFever, K. S. *et al*. Both Drosophila matrix metalloproteinases have released and membrane-tethered forms but have different substrates. *Sci. Rep.* **7**, 44560; doi: 10.1038/srep44560 (2017).

**Publisher's note:** Springer Nature remains neutral with regard to jurisdictional claims in published maps and institutional affiliations.

## Supplementary Material

Supplementary Information
