# Supplementary material for: Both Drosophila matrix metalloproteinases have released and membrane-tethered forms but have different substrates
Source: Sci Rep. 2017 Mar 16;7:44560. doi: 10.1038/srep44560 (PMC5353688; doi:10.1038/srep44560)

Supplemental Data: Original Western Blot Images

# Both Drosophila matrix metalloproteinases have released and membrane-tethered forms but have different substrates

Kimberly S. LaFever, Xiaoxi Wang, Patrick Page-McCaw, Gautam Bhave, and Andrea Page-McCaw

Original uncropped blots for Figure 2

A

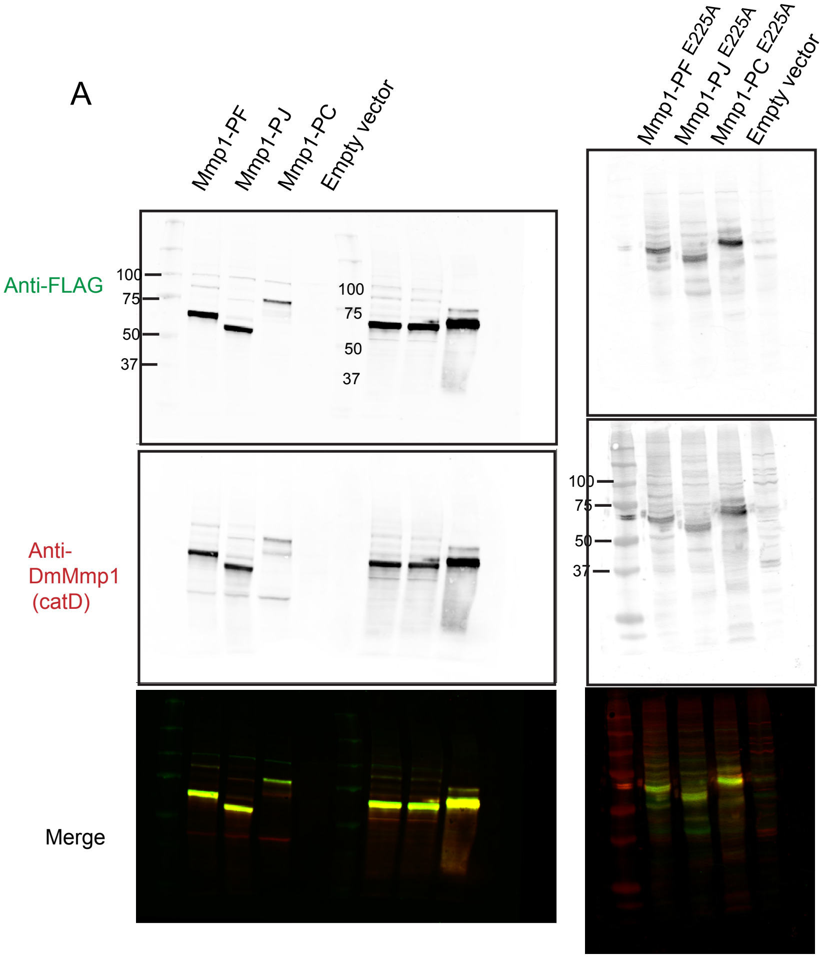

D

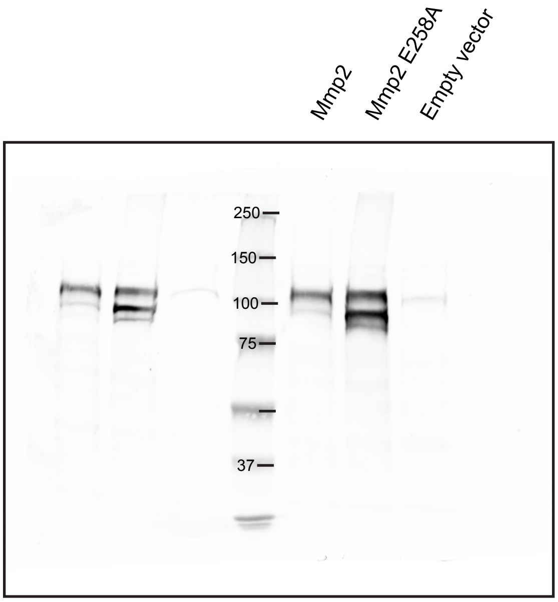

B

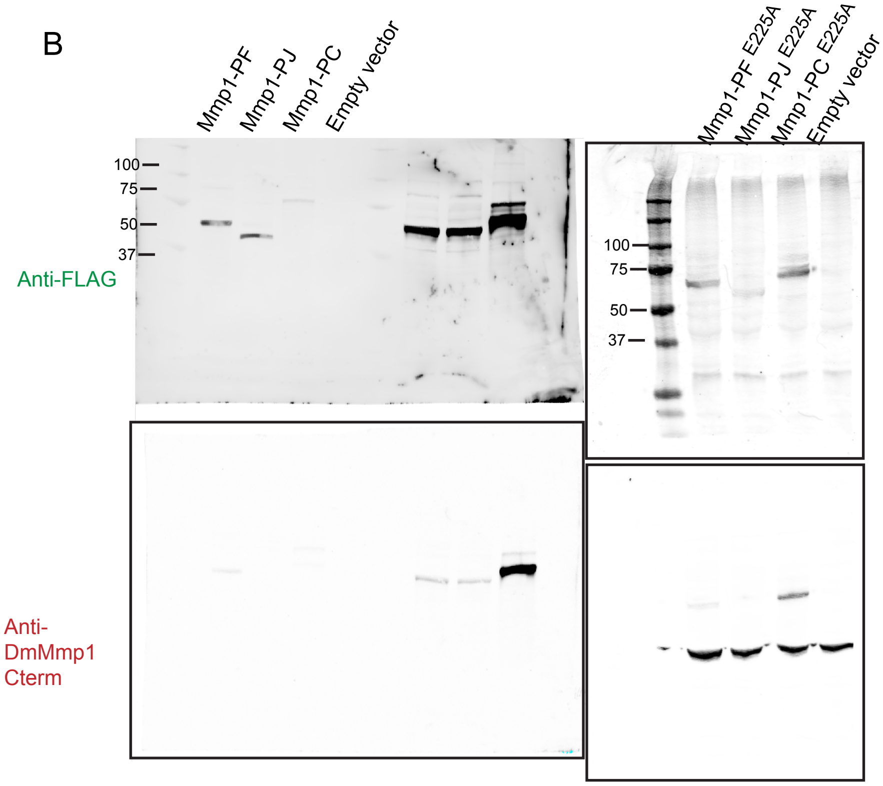

Original uncropped blots for Figure 3

A

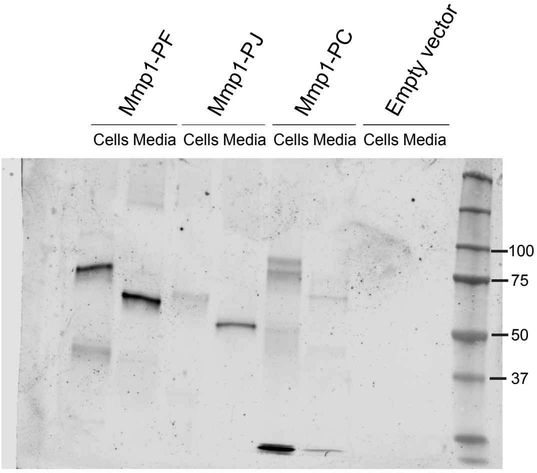

B

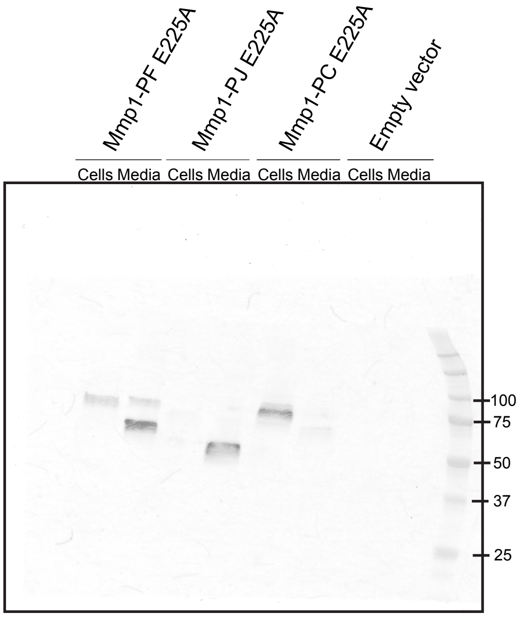

C

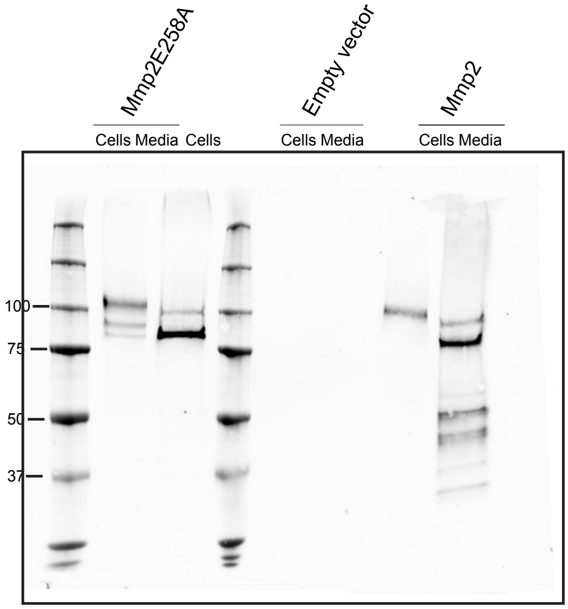

Original uncropped blots for Figure 5

A

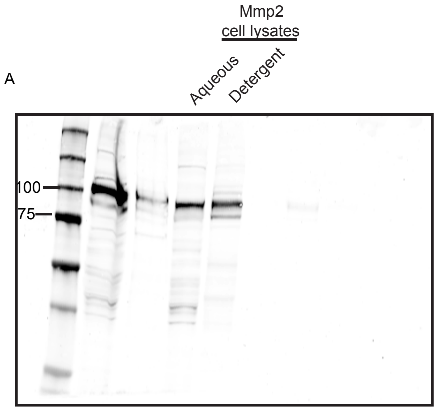

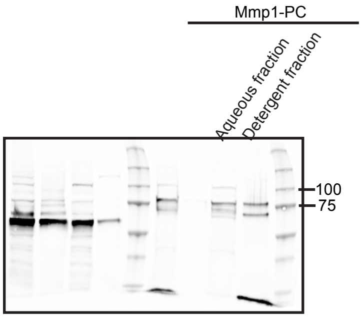

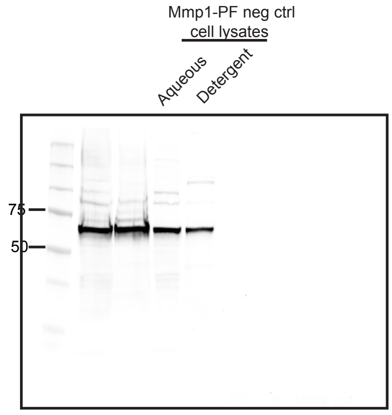

B

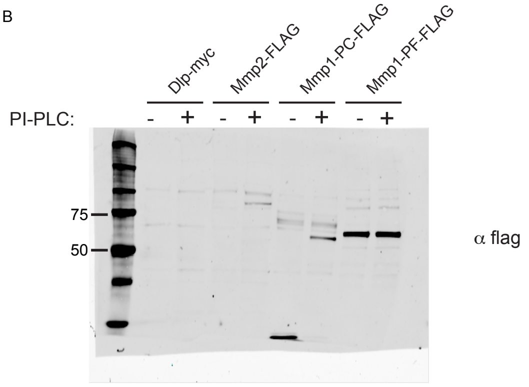

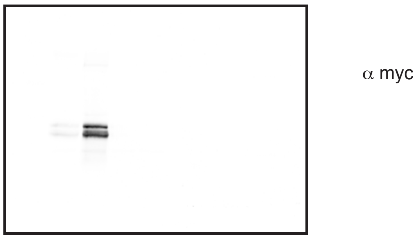

Original uncropped blots for Figure 7

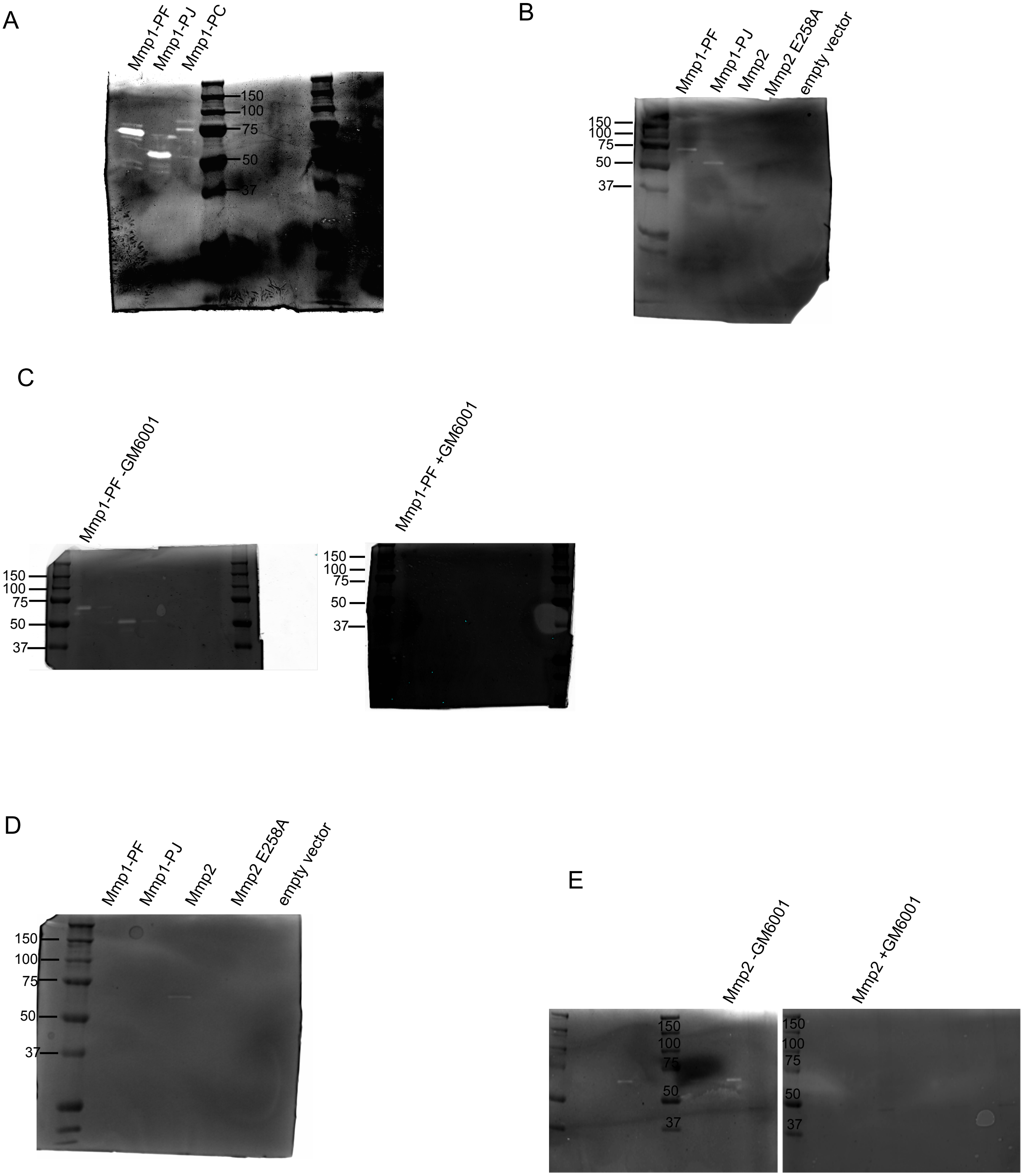

Supplement: Supplementary Information [file srep44560-s1.pdf]
